# Tort tradeoffs in cases of pesticide drift: A legal and economic analysis

**DOI:** 10.1371/journal.pone.0276418

**Published:** 2022-10-24

**Authors:** Nicholas Brown, Greg Colson, Matt Roessing

**Affiliations:** 1 School of Law, University of Georgia, Athens, Georgia, United States of America; 2 Department of Agricultural and Applied Economics, University of Georgia, Athens, Georgia, United States of America; 3 Terry College of Business, University of Georgia, Athens, Georgia, United States of America; Washington State University, UNITED STATES

## Abstract

Widespread pesticide drift issues ensued from the advent of dicamba-tolerant crop systems in the late 2010s, resulting in millions of acres of damaged farmland. Farmers who suffered drift-related losses in crop yield had to seek recovery in state courts. However, state courts varied in their approaches to drift lawsuits and remedies, if awarded, could include damage awards or injunctions. To demonstrate the need for a more transparent judicial process, this paper identifies three torts commonly advanced as causes of actions in drift cases and creates theoretic-game models to evaluate each tort’s impact on farmers’ decision-making and economic outcomes.

## 1. Introduction

Glyphosate-tolerant crops, commercially marketed as ‘Roundup Ready’ crops, were the first crop varieties to allow farmers to perform herbicide applications even after seedlings emerged from the soil [1]. After decades of widespread use, glyphosate-resistant weeds began to appear in increasing numbers. As a result, farmers desperately needed a new herbicide and herbicide-tolerant (HT) seed combination. Seed and pesticide manufacturers sought to satisfy this need with dicamba. Once used primarily as a preemergent, dicamba and dicamba-tolerant crops have now been implemented in soybean and cotton farming on a mass scale across the US. Dicamba had a noted history of volatility issues when it was used strictly as a preemergent, and its incorporation into dicamba-resistant crop systems only magnified this issue [2]. In 2018, dicamba drift was responsible for damage to approximately 4% of all soybean fields in the US, with Nebraska reporting damage to 8% of their state’s soybean fields alone [1]. Dicamba drift damage also extends to other crops. Although data on crops other than soybeans is relatively sparse, Missouri listed over 700 acres of peaches and nearly 20,000 tomato plants in a long list of crops and residential plant life damaged by dicamba drift in 2017 [3].

In its amended registrations for several dicamba formulations, the EPA indicated that there is a “lack of scientific consensus regarding the cause of these [off-target dicamba movement] incidents” [4]. This document, released before the 2018 growing season, cited input from various entities that listed suspected causes of dicamba drift including lack of adherence to application instructions, tank contamination, temperature inversions, and volatility. While dicamba drift’s causes are disputed, its effects are apparent and persistent losses from off-site dicamba movement demonstrate the need for an efficient system under which farmers can recover drift-related losses. For several decades, the interaction between Federal Insecticide, Fungicide, and Rodenticide Act (FIFRA) regulations, state case law, state right-to-farm acts, and the unique facts of each case have complicated farmers’ means of recovery. Further worsening matters, liability insurance policies for pesticide applications, which are often required for applicator licensure, are usually worded to avoid indemnification of third parties in instances of drift, regardless of whether the offending application violated any FIFRA-related regulations [5]. Additionally, crop insurance policies through the USDA Risk Management Agency likely will not help farmers suffering losses from drift [6].

As a result of these issues, farmers seeking to recover losses from pesticide drift typically sue in state civil courts, which vary from state to state in the causes of action they allow plaintiffs to allege, with trespass or negligence being the most common [7]. Depending on the cause of action, the plaintiff may have different burdens of proof and different available remedies. One might expect that, despite these differences, there is at least some cause of action in every state that allows a civil remedy for economic losses from pesticide drift, but this is not always correct [8]. The once relatively rare nature of pesticide drift suits means that many states have yet to address the issue, and the ad hoc nature of drift suits in these states adds to the difficulty of farmers’ attempts to recover losses [9]. This patchwork system of state judicial remedies for drift cases makes outcomes unpredictable, increasing business risks both for farmers whose crops are affected by drift and the pesticide applicators who face uncertain liability for their actions. The complex nature of these cases also increases the parties’ litigation costs [7].

Although a wide array of issues surrounding the adoption of pesticides have been explored in the academic literature, including issues of trade [10–12], land use [13, 14], and consumer acceptance [15–17], analysis of the legal system is more limited [18–21]. This paper addresses that gap in the literature by illustrating and comparing, in an economic framework, how each tort works in a trial setting when applied as a cause of action. The first section of this paper discusses the three major torts used in pesticide drift cases and their various legal aspects. Then, to formalize the tort comparison, the authors develop a game theoretic model of each tort to depict the decision-making process of the (1) pesticide applicator in their compliance decision and (2) the potentially harmed neighboring farmer in their decision to bring a lawsuit. The final section concludes the analysis by merging the results of each model with the legal characteristics of its respective tort and discussing the tradeoffs that farmers and policy-makers face under each tort model.

## 2. Legal discussion

This section discusses the three major causes of action that, under tort law, potentially could be raised by a farmer injured by another’s dicamba application. Regardless of the alleged tort, the harmed farmer must satisfy certain initial evidentiary requirements [7]. The first requirement is for a farmer to determine that pesticides have drifted onto his/her property. To meet this requirement, the farmer usually will call on crop experts to physically inspect fields where the drift allegedly occurred. The farmer will need to be prompt in calling on crop experts once any signs of drift-related damage emerge, as physical evidence of the pesticide on the surface of the crops can disappear quickly. Furthermore, many states require that farmers who have experienced losses due to pesticide drift notify their state agricultural agencies [22]. In states with such notification statutes, a farmer that detects drift damage but does not promptly notify their state agency is actually precluded from pursuing any action to recover their damages [23]. As noted in the models, state agencies that receive notification of drift damage will conduct their investigation separate from the harmed farmer and can levy their fees against the offending applicator if the application was deemed to be non-compliant.

A second requirement is that the affected farmer shows that the defendant applied the drifting pesticides (and not, e.g., a different farmer down the road). While the crop experts analyze the fields for any pesticide residue, the affected farmer also needs to secure the neighboring farms’ spray records, which the neighboring farmers must maintain in accordance with FIFRA [24]. Once collected, these records can be compared with recorded weather data and the findings of the crop experts to pinpoint neighbors suspected of spraying the pesticides that drifted [25].

A third requirement is that the farmer shows evidence of loss. As the fields become ready for harvest, the farmer needs to accurately estimate losses in yield due to the drift. This estimation will require aerial photographs of the damaged fields as visual estimates from the farmer will not be considered reliable [26]. The aggregation of this evidence will have to confirm that the losses incurred by the farmer are substantial, the threshold for which can vary from state to state.

The above is only a simplified version of the process that a farmer experiencing losses from drift damage might undergo. Additional steps might also involve collecting affidavits from the neighbor suspected of spraying the drifting pesticide and/or calling in additional crop experts to secure testimony on the extent of damage to the fields [7]. A farmer also needs to consider whether the crops showing signs of drift damage might instead be suffering from another issue (e.g., poor soil conditions, disease, etc.). Clearly, there are numerous possibilities for transaction costs that could be incurred in a pesticide drift suit, many of which need to occur before a would-be plaintiff can begin to estimate the likelihood of success and amount of recovery at trial. The accumulation of these costs throughout a lawsuit highlights the need for a transparent and efficient judicial process that minimizes other sources of inefficiency, increases predictability of legal outcomes, and promotes pre-trial settlements.

Moving onto the differences in each cause of action, each tort varies in what the plaintiff must prove and the available judicial remedies. Though each state court system has the power to interpret each tort in its own way, the significant influence of the Restatement of Torts has led to relative homogeny in how state courts define each tort [27]. And while definitions of trespass vary more from state to state than definitions of nuisance or negligence, only states with a certain interpretation of trespass can apply trespass to pesticide drift cases, a topic addressed in more detail later in this paper [28]. Therefore, to the extent states allow a particular cause of action in cases of drift, those states tend to require similar burdens of proof and apply similar remedies for that cause of action, allowing the authors to make economic comparisons between states.

### 2.1. Negligence

Negligence is defined as the failure to do something that a reasonably careful person would do or the doing of something that a reasonably careful person would not do [8]. For a plaintiff to succeed in a negligence suit, the plaintiff must show that the defendant had a duty of care that the defendant breached [29]. The plaintiff must prove that this breach in duty of care proximately caused damages to the plaintiff [30]. While proving duty of care and breach poses difficulty in many court cases, mandatory pesticide labeling under FIFRA and any applicable regulations from the state and local governments establish a clear duty of care for applicators to follow the laws and label instructions regarding application [31].

Before a pesticide can be registered for sale, the EPA must determine that applications of said pesticide that adhere to the label do not cause “unreasonable adverse effects on the environment” [32]. This key and controversial phrase in FIFRA enables the EPA to use a cost-benefit analysis in their registration decisions [33]. This cost-benefit analysis considers economic, social, and environmental aspects of the pesticide, meaning that a pesticide may be approved despite certain costs if other benefits outweigh those costs [34]. Once a pesticide is registered under FIFRA, applicators must apply in strict accordance with the EPA-approved label [35].

While states and local governments are preempted under FIFRA from enacting regulations concerning labeling and packaging, the Supreme Court in *Bates v*. *Dow Agrosciences LLC* (2005) confirmed the “State’s broad authority to regulate the sale and use of pesticides” [36 p446]. Before *Bates*, most state and local pesticide regulation was conservatively written as courts had historically struck down more expansive regulations from states and local governments. Courts justified these decisions because such regulations ‘induced’ pesticide manufacturers to modify their labels and packaging. Those state and local regulations were thus preempted under FIFRA’s preemption clauses concerning labeling and packaging. In *Bates*, the Supreme Court ruled against the inducement test in use by lower courts, citing that “the inducement test is not supported by either text or the structure of the statute” [36 p446]. The Supreme Court’s ruling in *Bates* set forth a narrower definition of the preemption clause in FIFRA and opened the door for unprecedented state and local pesticide regulation. After the 2017 growing season in which dicamba drift became widespread, many state agricultural agencies utilized their expanded regulatory capabilities to establish cutoff dates for dicamba applications [37–39]. State and local pesticide regulations such as these cutoff dates serve as the second half of the duty of care for pesticide applicators to follow.

Several states, including Michigan, Minnesota, and South Carolina, do not interpret trespass law as applicable to drift and have “right-to-farm” acts that prohibit drift-related nuisance suits, making negligence the default cause of action in those states [7]. This distinction means that states not classified as using a trespass or nuisance system require plaintiffs to prove that the applicator violated a regulation and subsequently caused the pesticide to drift. In cases where the applicator did not comply with the pesticide label or application regulations, plaintiffs receive monetary damages equivalent to their losses [39]. In cases where the applicator followed all instructions and regulations, negligence offers no means of recovery for farmers who have taken drift-related losses [8]. It is worth noting that, with dicamba, the EPA responded to the widespread drift issues with an amended registration that updated labeling restrictions “to further minimize the potential for off-site movement” [4]. This labeling update implies that at least some of the 2017 drift incidents may have been caused by applications that, at the time, were compliant.

It also should be noted that, even in states with a trespass or nuisance system, negligence can still be applied in cases where the applicator failed to abide by all necessary regulations [40, 41]. In these instances, plaintiffs can file both negligence and trespass/nuisance claims [42]. While the plaintiff likely would not receive more than the amount of their actual damages, the defendant might be subject to additional fines levied by their state’s agricultural agency and other punishments related to their pesticide licensing [35, 43, 44].

### 2.2. Trespass

Trespass is defined as an invasion of the interest in the exclusive possession of another person’s land, as by entry on it [44]. Trespass is not limited to a person physically stepping onto someone else’s land; it can also be applied where a person causes the entry of an object onto another person’s property. Historically, trespass was limited to cases with tangible invasions (e.g., person wrongfully entering the property, person wrongfully leaving a vehicle on the property) [45]. Courts viewed tangible invasions through the dimensional test, which is the traditional common law rule that requires an invasion of land by a physical, tangible object [7].

While the dimensional test met the needs of courts throughout most of history, the discovery of microscopic particles led to court arguments that the invasion of particulate matter (e.g., smoke, dust, odors, etc.) onto other properties constituted a trespass [46]. Courts in states such as Alabama and Washington accepted this argument but attached the requirement for the plaintiff to suffer ‘substantial damages’ from the ‘intangible invasion’ [28]. Without the requirement for substantial damages, courts recognized the potential for trivial lawsuits as “every property in the State would have a cause of action against any neighboring industry which emitted particulate matter into the atmosphere, or even a passing motorist, whose exhaust emissions come to rest upon another’s property” [46 p529].

Other states rejected the application of trespass theory to intangible invasions. Some of those states found the ‘substantial damages’ compromise incompatible with trespass law’s basic assumptions about private property rights. For example, in *Adams v*. *Cleveland-Cliffs Iron Co*. (1999), the Court of Appeals of Michigan stated that “the law should not require a property owner to justify exercising the right to exclude” [47 p221]. Therefore, rather than allowing trespass to apply to intangible invasions on a case-by-case basis, the court barred the application of trespass to such claims. Similarly, the Supreme Court of Minnesota in *Johnson v*. *Paynesville Farmers Union Coop*. *Oil Co*. (2012) stated that the inclusion of intangible invasions under trespass theory “conflicts with our precedent defining the elements of trespass” [30 p703]. In the same case, the Supreme Court of Minnesota would go on to note that if an intangible invasion causes substantial damages as required in other states for trespass, then “the emission will also likely be an unreasonable interference with plaintiff’s use and enjoyment of his land, and therefore constitute a nuisance” [30 p704].

For trespass, the measure of monetary damages is calculated as the value of the use of the property [46]. In pesticide drift cases, courts would likely use the value of expected future profits from the damaged crops [48]. Furthermore, trespass theory places a sacred value on private property rights. As such, injunctions are far more likely to be issued with trespass than with nuisance, even in the circumstances involving a compliant applicator. In *Bradley v*. *American Smelting and Refining Co*. (1985), the Washington Supreme Court discussed the differences between nuisance and trespass. There, the Court stated in part that “the principal difference in theories is that the tort of trespass is complete upon a tangible invasion of plaintiff’s property…the protection of the integrity of his possessory interests might justify the injunction” [28 p787]. Other courts that adopted the use of trespass in cases of pesticide drift have also displayed a willingness to enjoin applicators. In *Macalpine v*. *Hopper* (2012), a Colorado district court issued an injunction against a pesticide applicator accused of performing applications resulting in drift. Here, the applicator had been given a brief explanation on how to apply the pesticides by local government officials, but there were gaps in their instructions to the applicator [49]. Using the little instruction he was given, the applicator did perform non-compliant applications in the beginning, but he received the proper training and henceforth only applied the pesticide in a compliant fashion. Still, the court issued an injunction against the applicator, noting that “the public has a strong interest in protecting and preserving property rights from invasions by others” [49 p9].

### 2.3. Nuisance

With respect to the courts that refused to allow intangible invasions under trespass theory, the common justification was that doing so “blurs the line between trespass and nuisance” [30 p704]. An individual can be held liable for a nuisance if his/her actions cause the invasion of another’s interest in the private use and enjoyment of their land [50]. For this invasion to be actionable in court, the invasion must be either intentional and unreasonable or unintentional but due to negligent conduct or ultrahazardous activity. The Restatement (Second) of Torts also calls on courts to consider the utility of the offending activity versus the gravity of the harm that it is causing. Therefore, nuisance suits can be successful in court even if they pursue otherwise lawful activities. Dicamba applications that adhered to the label and other regulations but still drifted are thus the textbook example of a nuisance [51].

The Supreme Court of Nebraska in *Hall v*. *Phillips* (1989) heard arguments concerning atrazine drift from a corn field to a bean field [52]. The applicator was found to be free from negligence during the pesticide application and asserted that the drift resulting from severe winds resulted as an Act of God. The lower court had granted summary judgment in favor of the applicator, finding that the drift did not qualify as a nuisance. The Supreme Court of Nebraska found that the lower court erred in awarding summary judgment. Regarding the lack of negligence during the application, the Supreme Court of Nebraska stated that “an invasion or interference which is substantial may result in equitable liability for a private nuisance and consequent damages, regardless of the reasonableness of the interference” [52 p145]. While Nebraska’s right-to-farm act was never addressed in *Hall*¸ it is worth noting that their right-to-farm act only protects farming practices that would not have been considered a nuisance when the farm commenced operations [53]. Such language is relatively rare in other states’ right-to-farm acts, and therefore right-to-farm legislation can be relevant in many nuisance cases, as discussed below [54, 55].

The payment of monetary damages is common in nuisance cases [56]. While injunctions are also permitted in nuisance cases, nuisance law’s standard of reasonableness should support applicators who operate in good faith, especially when they satisfy their duty of care in all applications [57]. The Supreme Court of Minnesota reinforced this principle in *Johnson v*. *Paynesville Farmers Union Coop*. *Oil Co*. (2012), where the Court stated in part that “the defendant’s liability for nuisance is determined by balancing the ‘social utility of the defendant’s actions with the harm to the plaintiff’” [30 p706]. With modern agriculture being so pesticide-dependent, it would be challenging for a plaintiff to convince a judge that the reasonable solution to otherwise lawful pesticide applications that drift is to completely enjoin the applicator from further use of the pesticide. Instead, monetary damages would almost always be the result in pesticide drift suits involving a compliant applicator, which would require the applicator to absorb the external costs of their applications without completely shutting down their farm [52]. But in the case of a non-compliant applicator, a court might award an injunction [49].

There is not a wide body of case law concerning the application of nuisance law to pesticide drift because, in many states, right-to-farm acts prevent most nuisance suits against farms [58]. Though each state’s right-to-farm act is different, they all share the overriding concept that a property owner’s agricultural activities should not be considered a nuisance as locality changes. For historical context, legislatures adopted these laws in response to numerous nuisance suits filed against farms in the late 20^th^ century. These farms, which had long been in operation in historically rural areas, were now enveloped by urban sprawl. Not used to the sights and smells of swine operations and chicken houses, their new neighbors filed nuisance suits to shut down these farms. To prevent these suits, legislatures passed right-to-farm acts. Frequently, states would write that the express purpose of their right-to-farm act was to protect the state’s agricultural industry [42]. While these laws were not written with pesticide drift in mind, their language often preempts nuisance claims for drift. Consequently, there are no states with sufficient case law or specially drafted right-to-farm laws that would allow the categorization of that state as having a nuisance system.

## 3. Tort models in an economic framework

With pesticide drift, economic modeling can become quickly complicated by various agricultural and legal factors that ultimately complicate the model’s tractability. Per economic custom, certain assumptions have to be made. While some assumptions are discussed alongside the tort models, five assumptions with lengthier descriptions need to be addressed before laying out the models. The first key assumption will involve the technical language used by the judge in writing the injunctions that appear in both the trespass and nuisance models. In order to focus on the most relevant outcomes of a trial, only permanent injunctions will be used in the models. Permanent injunctions forever govern the actions of the enjoined party (here, the enjoined applicator) after the trial. In contrast, preliminary injunctions (issued independently of permanent injunctions) are effective only during the trial. Thus, preliminary injunctions carry far less incentive for the decision-making of either side and are appropriately excluded from the models. Moreover, the permanent injunction will prohibit the enjoined farmer from future applications of the pesticide that drifted. Since permanent injunctions are written at the discretion of the court, we assume that the judge will rely on the available agriscienctific data in the technical language of the injunction [49]. Current research suggests that pesticides can drift over a mile under certain conditions [26]. Therefore, we assume that a judge seeking the most equitable solution for both sides would permanently enjoin the offending applicator from future applications of the pesticide that drifted while still allowing them to apply other pesticides.

The second assumption made when establishing the models is that no settlements occur during the pre-trial process or the trial itself. Much like the prior assumption that excluded preliminary injunctions, the assumption that no settlements occur runs counter to how most drift incidents will develop (i.e., both parties, in reality, would prefer to settle before the trial’s conclusion to minimize legal costs). However, the assumption that no settlements occur is vital to the model’s overall effectiveness and policymakers’ ability to draw valuable insight from it. By allowing a hypothetical drift incident to develop into a full trial, each model can be effectively compared with respect to its effects on farmer decision-making. Given the ability to compare models, policymakers can make their conclusions about the usefulness of negligence, trespass, and nuisance claims in efficiently and effectively resolving drift disputes.

The third assumption used in forming the models is that the plaintiff and defendant will incur equivalent legal expenses during the trial. This assumption generally holds in trial settings where the plaintiff succeeds (as noted in the fourth assumption, plaintiff success is guaranteed throughout all trial scenarios). Given plaintiff success, the plaintiff’s attorney, who traditionally operates on a contingency basis, will charge the plaintiff a percentage fee based on the amount of monetary damages. Plaintiffs’ attorneys often charge 30–40% of their client’s award in addition to any costs incurred during the trial, such as crop experts being used as witnesses [59]. Though plaintiff success is guaranteed in the models, the fee structure of plaintiffs’ attorneys means that most will not accept a case unless a certain amount of damages can reasonably be expected to be won. Despite these differences in fee structures, the plaintiff and defendant, whose respective attorneys will spend several months trying to outmatch the other with corresponding expert witnesses and evidence, will eventually pay similar legal fees.

The fourth assumption is perfect information concerning the neighboring farmer’s ability to accurately identify drift damage, the suspected responsible applicator, and the compliance decision of said applicator. Without this assumption, a wide range of problems could hamper the models. These problems could include farmers who allege drift damage when the damage is related to drought or farmers who allege pesticides drifted off of one neighbor’s field when they actually drifted off of another neighbor’s field. In reality, most farmers are familiar with all relevant circumstances and information to avoid these issues, making this assumption reasonable. Since plaintiffs will know when the facts of the case support a favorable ruling, trials will only occur where the plaintiff is guaranteed success. In other words, all trials that take place in the model will result in a favorable ruling for the plaintiff because the plaintiff would not file suit if they expected to lose.

The fifth and final assumption is that the defendant will not be required to pay punitive damages under any modeled scenario. Punitive damages exist within tort liability to enable the court to punish the defendant for any wanton and willful misconduct [60]. In cases of pesticide drift, wanton and willful misconduct would, at the bare minimum, require non-compliance from the applicator. Of all the relevant cases cited in this paper, very few of the plaintiffs sought punitive damages [30, 40, 61]. Of these limited cases, plaintiffs’ requests for awards of punitive damages were repeatedly denied at the trial court level. Given this precedent, punitive damages are excluded from the models.

Given the details of those five assumptions, it is now appropriate to discuss the models of each tort. To formally compare and contrast the effect of different legal structures on (a) the incentive to comply with label requirements, (b) economic outcomes for pesticide applicators and potential harmed parties, and (c) the prevalence of lawsuits when damages occur, we develop a simple game theoretic model. We consider a setting with two players, the applicator (i.e., the defendant in the event of a lawsuit) and the neighboring farm (i.e., the plaintiff in the event of a lawsuit). In this game, the applicator moves first and decides whether to *Comply* or *Not Comply* with the pesticide’s label. Applicator profit under compliance is Δ^*C*^ and non-compliance is Δ^*NC*^, where Δ^*NC*^ ≥ Δ^*C*^. Applicator profit under non-compliance is higher because the applicator is no longer constrained to the application cutoff dates, weather restrictions, buffer zone rules, among other rules imposed by the label [38]. Without these constraints, the non-compliant applicator can spray more pesticide over larger quantities of cropland.

Conditional on the compliance decision, nature determines if and to what degree damages occur to the neighboring farm. We consider a setting where three potential damage levels may occur on the neighboring farm: high damages $\bar{D}$, low damages $\underline{D}$, or no damages where $\bar{D}>\underline{D}>0$. Letting Π denote the neighboring farm profit in when no damages occur, this implies in the absence of legal remedies the neighboring farm profit would be $\prod -\bar{D},\;\prod -\underline{D}$, or Π depending upon the degree of damage. The probability of these three damages levels occurring are, respectively, ${\bar{P}}^{C},{\underline{P}}^{C}$, and $1-{\bar{P}}^{C}-{\underline{P}}^{C}$ if the applicator complies and ${\bar{P}}^{N}{}^{C},{\underline{P}}^{NC}$, and $1-{\bar{P}}^{N}{}^{C}-{\underline{P}}^{NC}$ if the applicator does not comply. The probability of damages is assumed to be greater under non-compliance, ${\bar{P}}^{N}{}^{C}>{\bar{P}}^{C}$ and ${\underline{P}}^{NC}>{\underline{P}}^{C}$, hence the probability of no damages is greatest under compliance. Once nature determines what level of damages occurs conditional on the compliance decision by the applicator, the neighboring farm decides whether to bring a lawsuit. We assume if a lawsuit is filed, both the plaintiff and defendant bear legal expenses denoted by *L*. To distinguish between levels of damages sufficient for a plaintiff and legal team to be willing to bring a lawsuit, without loss of generality we assume $\bar{D}>L>\underline{D}$. This assumption implies, in the absence of other benefits from filing a lawsuit, a plaintiff may be willing to file a lawsuit in the event of high damages but not if low damages occur. As would be expected, the neighboring farmer optimally would not file a frivolous lawsuit in the event of zero damages.

We denote potential fines and penalties levied by the EPA and state agencies on the plaintiff as *F*. Notably, these fines, if issued, are the result of a state agency-led investigation that is conducted independently of any trial proceedings and the money collected from it goes to the state agency alone (i.e., none to the harmed neighboring farmer). Per notification statutes in many states, the model assumes that the state is notified by the neighboring farmer as required to pursue their losses in court, except in instances where no damages occur since the neighboring farmer would not have any crop damage to present to the state. Finally, in the event the court grants an injunction against the applicator, we denote the value of an injunction against all future applications of the drifting pesticide on the applicator’s farm as *E*. For simplicity we assume the legal expenses, *L*, and the value or cost of an injunction, *E*, are the same for both the plaintiff and defendant and are independent of the level of damages. Profits for both parties that are presented in bold denote the neighboring farmer’s optimal strategy given an observed level of damages. Absence of bold for a terminal node denotes an outcome without a dominant strategy on the part of the neighboring farm without further assumptions, which will be discussed later in detail.

### 3.1. Negligence model

**Fig 1:**
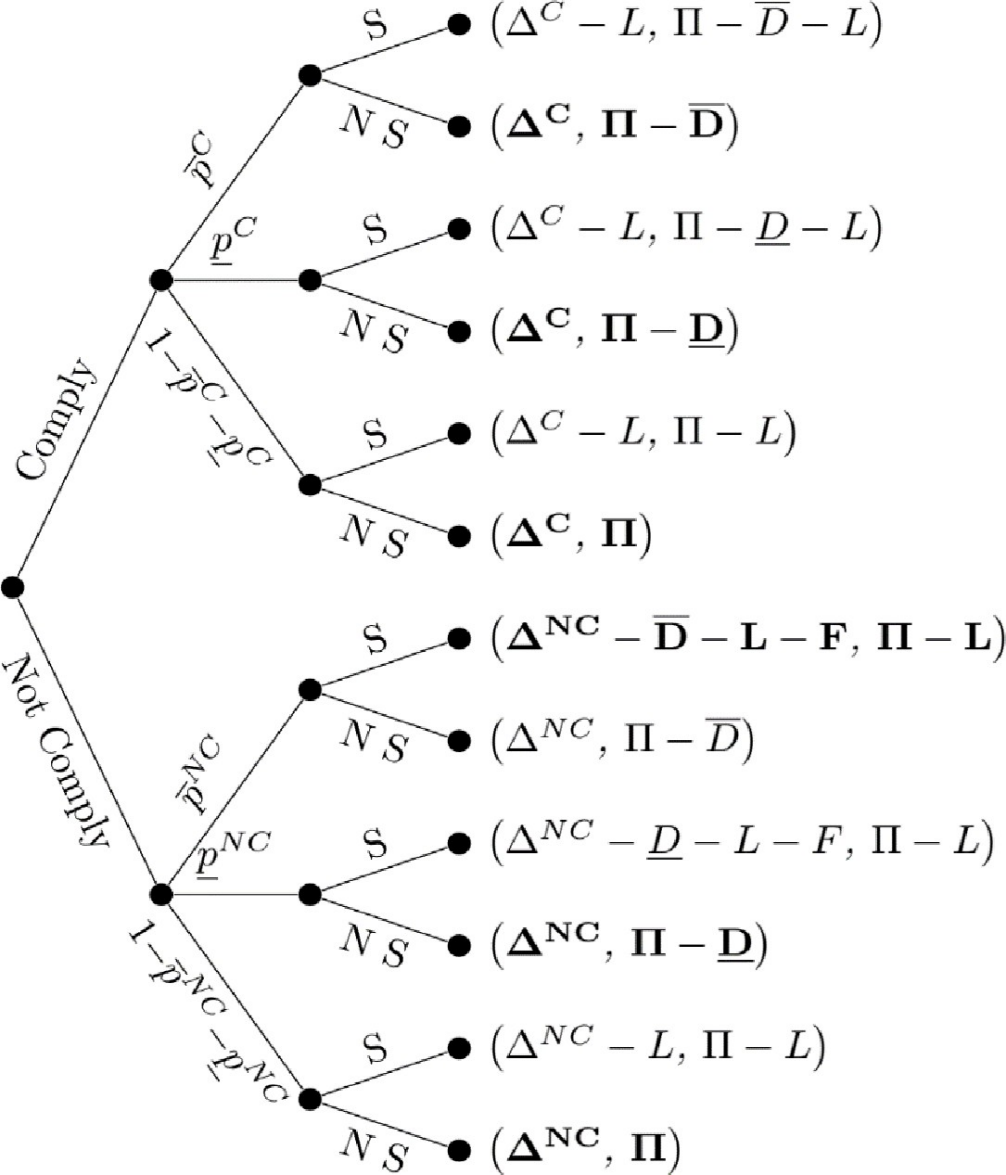
Negligence. Applicator and neighboring farm profits in parentheses. Profits for both parties that are presented in bold denote the neighboring farmer’s optimal strategy given an observed level of damages. Absence of any bold for a terminal node denotes an outcome without a dominant strategy on the part of the neighboring farm without further assumptions.

Given this game theoretic structure, Fig 1 presents a compliance-lawsuit game under a negligence system. As with all the models, it is assumed that the neighboring farmer knows whether the applicator was compliant or non-compliant. While observing high damage to crops is insufficient to determine compliance, a farmer should be able to satisfy the burden of proof if the fourth assumption holds. Under negligence, the lawsuit is only successful if the applicator was not compliant. Further, given the absence of potential injunctions against further activity, lawsuits will only be filed when high damages, $\bar{D}$, occur. Given that the plaintiff will only file a lawsuit if high damages occurs and (in this perfect information setting) the applicator was non-compliant, the expected profit for the applicator under compliance is Δ^*C*^. Therefore, expected profit if the applicator does not comply is $\Delta^{NC}-{\bar{P}}^{NC}(\bar{D}+L+F)$. The applicator will weigh expected profit by complying or not complying with pesticide label requirements and hence will not comply if:

$$\Delta^{NC}-\Delta^{C}>{\bar{P}}^{NC}(\bar{D}+L+F)$$

### 3.2. Trespass model

**Fig 2:**
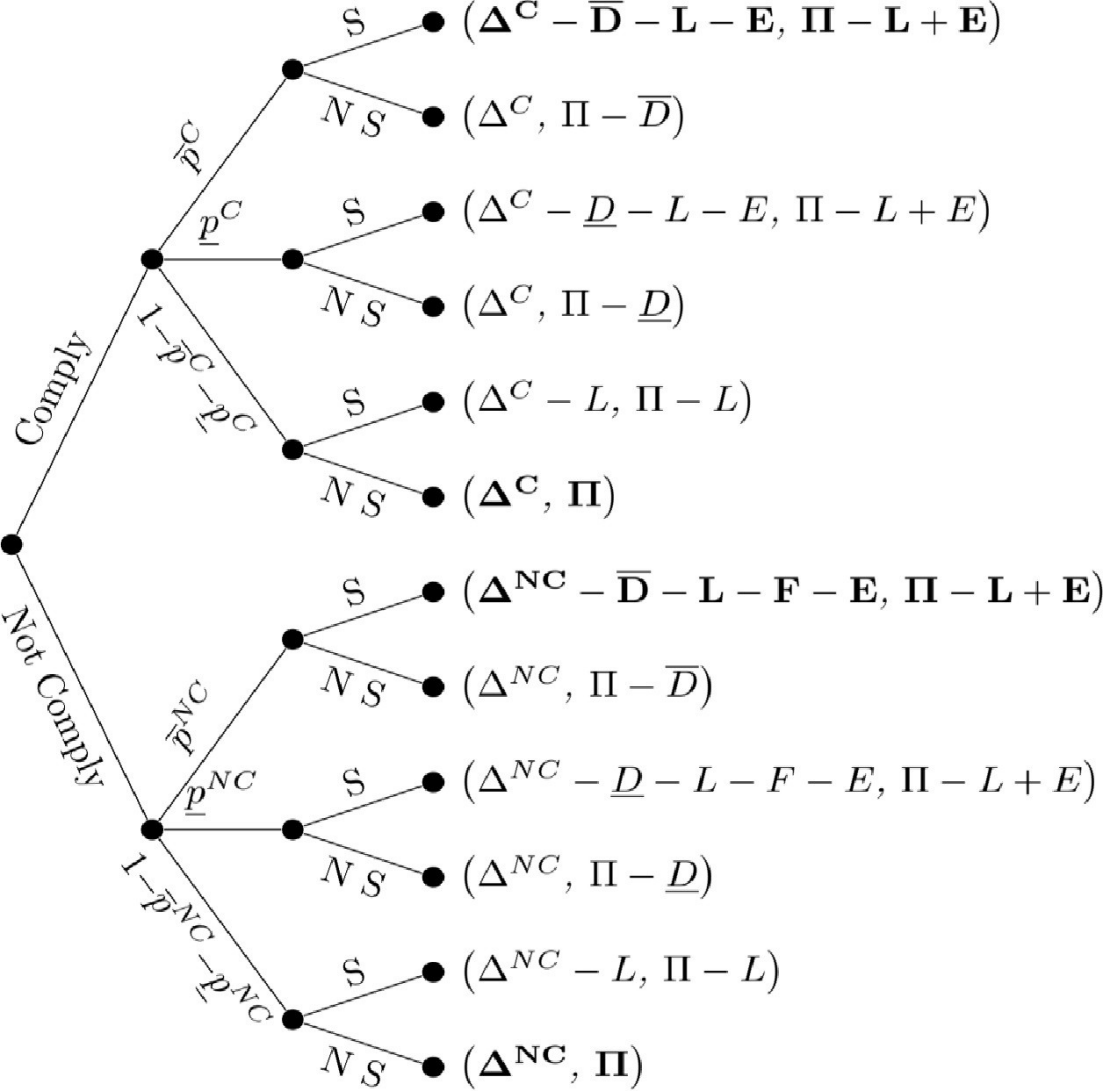
Trespass. Applicator and neighboring farm profits in parentheses. Profits for both parties that are presented in bold denote the neighboring farmer’s optimal strategy given an observed level of damages. Absence of any bold for a terminal node denotes an outcome without a dominant strategy on the part of the neighboring farm without further assumptions.

Trespass is distinguished from both negligence and nuisance by the fact it produces the same outcomes for both the neighboring farmer and applicator regardless of compliance, except for the inclusion of EPA and state-levied fines, *F*, in the payoff for the non-compliant applicator. As can be seen in Fig 2, regardless of whether the applicator is compliant or not, the neighboring farm will sue if high damages, $\bar{D}$, occur. Given that under trespass the neighboring farm can recover damages, regardless of compliance, when low damages, $\underline{D}$, occur, the decision whether to sue in this setting depends upon whether $E+\underline{D}>L$ or not. If $E+\underline{D}>L$, then the neighboring farm will bring a lawsuit when low damages occur, else it would be suboptimal to sue. This result is similar to the outcome yielded in the nuisance model’s non-compliance setting, but distinctly under trespass this result occurs in both the compliance and non-compliance setting.

From the perspective of the applicator, we can summarize the expected profit from compliance and non-compliance, conditional on the relative value of the injunction as: $\Delta^{C}-{\bar{P}}^{C}\left(\bar{D}+L+E\right)$ and $\Delta^{NC}-{\bar{P}}^{NC}\left(\bar{D}+L+F+E\right)$ when $E+\underline{D}<L$. When the value of the injunction is sufficiently large to trigger lawsuits under low damages, $E+\underline{D}>L$, applicator compliance and non-compliance expected profits are $\Delta^{C}-\left({\bar{P}}^{C}+{\underline{P}}^{C}\right)\left(L+E\right)-{\bar{P}}^{C}\bar{D}-{\underline{P}}^{C}\underline{D}$ and $\Delta^{NC}-\left({\bar{P}}^{NC}+{\underline{P}}^{NC}\right)\left(L+F+E\right)-{\bar{P}}^{NC}\bar{D}-{\underline{P}}^{NC}\underline{D}$. Hence, we can express the conditions under which the applicator will not comply as:

$$\begin{array}{cc} \Delta^{NC}-\Delta^{C}>\left({\bar{P}}^{NC}-{\bar{P}}^{C}\right)\left(\bar{D}+L+E\right)+\left({\underline{P}}^{NC}-{\underline{P}}^{C}\right)\left(\underline{D}+L+E\right)+{\bar{P}}^{NC}\left(F\right)+{\underline{P}}^{NC}\left(F\right) & if\;E+\underline{D}>L\\ \Delta^{NC}-\Delta^{C}>\left({\bar{P}}^{NC}-{\bar{P}}^{C}\right)\left(\bar{D}+L+E\right)+{\bar{P}}^{NC}\left(F\right) & if\;E+\underline{D}<L \end{array}.$$

### 3.3. Nuisance model

**Fig 3:**
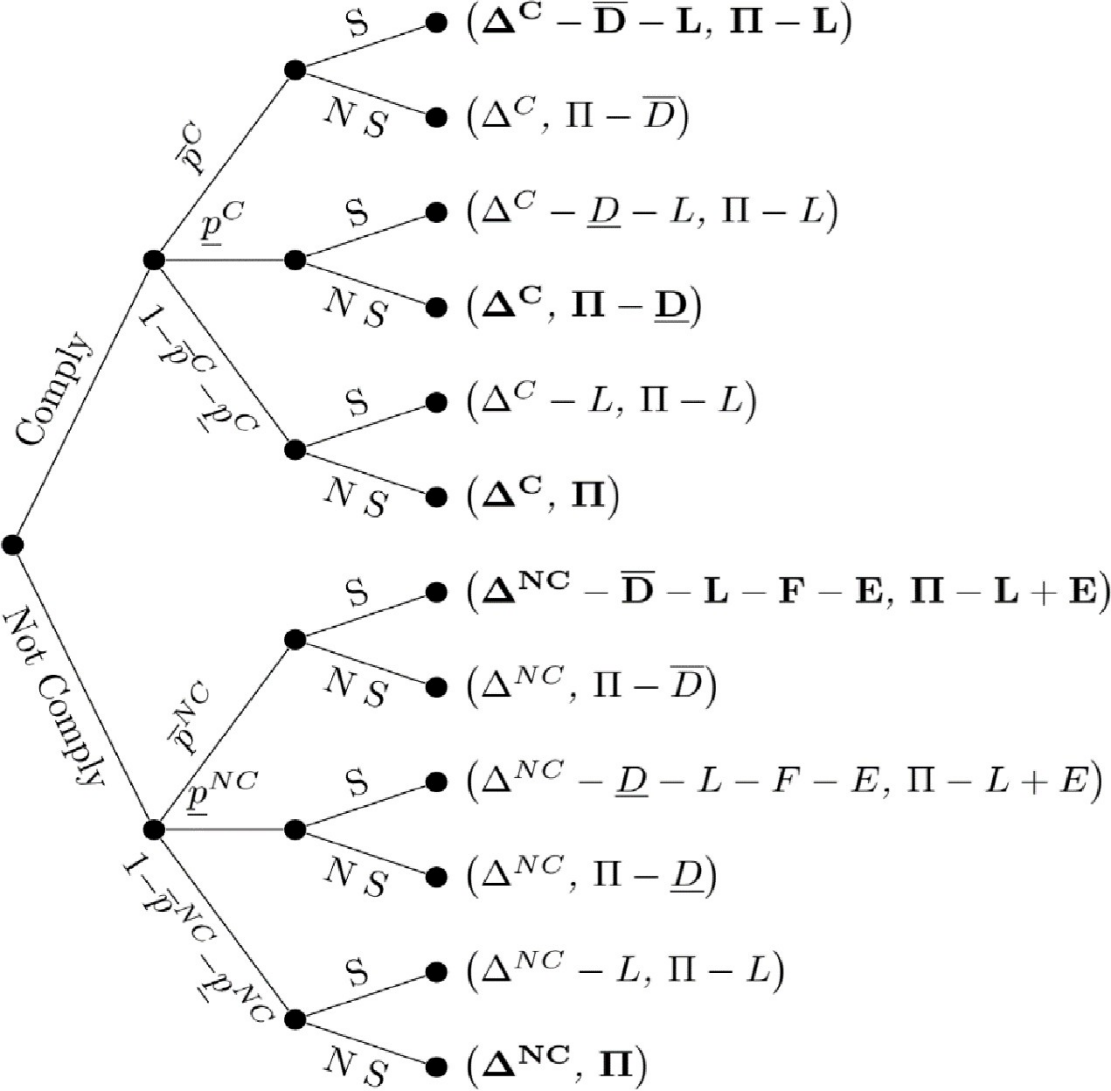
Nuisance. Applicator and neighboring farm profits in parentheses. Profits for both parties that are presented in bold denote the neighboring farmer’s optimal strategy given an observed level of damages. Absence of any bold for a terminal node denotes an outcome without a dominant strategy on the part of the neighboring farm without further assumptions.

Given this game theoretic structure, Fig 3 presents the applicator-neighboring farm game in extensive form for the case of nuisance. Under nuisance, the neighboring farm will file a lawsuit whenever high damages $\bar{D}$ occur, regardless of applicator compliance. The decision to file a lawsuit when low damages, $\underline{D}$, occurs hinges on two factors: whether the applicator was compliant and the relative value of the injunction to the neighboring farmer. Assuming the potential plaintiff is able to distinguish whether the applicator was compliant or not, optimally he will not file a lawsuit against a compliant applicator who caused low damages. If the applicator was not compliant, the neighboring farm will sue if the value of the recouped damages and the potential injunction outweighs the cost of bringing the lawsuit. Specifically, a lawsuit will be filed if $E+\underline{D}>L$, and not otherwise. Given that the plaintiff will always file a lawsuit if high damages occurs, the expected profit for the applicator under compliance is $\Delta^{C}-{\bar{P}}^{C}\left(\bar{D}+L\right)$, profit under compliance less the loss in the event of high damages. Under non-compliance, a lawsuit will also be filed if high damages occur and potentially under low damages conditional on the value of the potential injunction. Hence, expected profit for a non-compliant applicator is $\Delta^{NC}-\left({\bar{P}}^{NC}+{\underline{P}}^{NC}\right)\left(L+F+E\right)-{\bar{P}}^{NC}\bar{D}-{\underline{P}}^{NC}\underline{D}$ if $E+\underline{D}>L$ and $\Delta^{NC}-{\bar{P}}^{NC}\left(\bar{D}+L+F+E\right)$ if $E+\underline{D}<L$. The applicator will weigh expected profit by complying or not complying with pesticide label requirements and hence will not comply if:

$$\begin{array}{cc} \Delta^{NC}-\Delta^{C}>{\bar{P}}^{NC}\left(\bar{D}+L+F+E\right)-{\bar{P}}^{C}\left(\bar{D}+L\right) & if\;E+\underline{D}<L\\ \Delta^{NC}-\Delta^{C}>\left({\bar{P}}^{NC}+{\underline{P}}^{NC}\right)\left(L+F+E\right)+{\bar{P}}^{NC}\bar{D}+{\underline{P}}^{NC}\underline{D}-{\bar{P}}^{C}\left(\bar{D}+L\right) & if\;E+\underline{D}>L \end{array}$$

That is, the applicator will not comply if the incremental gain in profit from not complying, Δ^*NC*^ − Δ^*C*^, outweighs the expected loss if high damages occur. As can be seen, when the value of the injunction is sufficiently low such that the neighboring farm will not bring a lawsuit in the event of low damages ($E+\underline{D}<L$), the applicator has greater incentive to be non-compliant because the applicator does not face the risk of a legal challenge unless high damages occur.

### 3.4. Implications of models

Comparing all three legal structures–negligence, nuisance, and trespass–reveals multiple implications for the incentive of applicators to comply with regulations, prevalence of lawsuits, and profit of neighboring farmers and applicators. Under negligence, lawsuits will only be filed when the applicator is not compliant and high damages occur. Of the three systems, negligence offers the highest incentive to comply and the lowest expectancy for lawsuits. As a result, negligence offers the lowest expected profit for neighboring farmers and the highest expected profit for applicators, relative to all other models.

Trespass, on the other hand, offers a much different outcome for neighboring farmers and applicators. Under a trespass system, lawsuits are filed when *E* + *D* > *L*, regardless of the level of damages (assuming it is greater than zero) and applicator compliance. The large disregard for compliance under trespass results in the lowest incentive for the applicator to comply and the highest expectancy for lawsuits. Due to their greater ability to recover damages and enjoin surrounding applicators, neighboring farmers under trespass have their highest expected profit of all three models. For the same reasons, applicators have their lowest expected profit under trespass.

Relative to negligence and trespass, nuisance provides a middle ground for the comparisons of expected number of lawsuits, incentive for applicator compliance, and expected profits for both parties. Neighboring farmers under a nuisance system will sue a compliant applicator only when damages are high but will sue a non-compliant applicator when *E* + *D* > *L*, regardless of the level of damages (assuming it is greater than zero). This decision-making process for the neighboring farmers leads to an incentive for applicator compliance under nuisance that exceeds the incentive under trespass but is less than the incentive under negligence. Consequently, the expected profit for neighboring farmers is higher under nuisance than under negligence but lower than under trespass. The opposite is true for applicators under a nuisance system, who face a lower expected profit than under negligence and a higher expected profit than under trespass.

### 3.5. Numerical illustration

Although each case of damage from pesticide drift is unique due to differences in crops involved, scale and magnitude of damages, state-specific fines, etc., and the probability of damages occurring under compliance and non-compliance are a point of contentious debate, to illustrate the differences in the incentive for applicators to comply with labeling requirements under different legal settings, we present two numerical examples. First, consider a setting mirroring statements by the EPA that the probability of damages from an applicator complying with labeling requirements is zero (${\bar{P}}^{C}={\underline{P}}^{C}=0$, i.e., damages only occur when the applicator is non-compliant. Consider an applicator and neighboring operation growing similar crops with the same value under compliance Π = Δ^C^. Under non-compliance potential damages are 50% and 10% of the crop value ($\bar{D}=0.5\;\prod$ and $\underline{D}=0.1\;\prod$). Legal fees are assumed to have the traditional rate structure $L=0.3\bar{D}$, fines are equal to the lower level of damage ($F=\underline{D}$), and the value of an injunction is $\bar{D}$.

**Fig 4:**
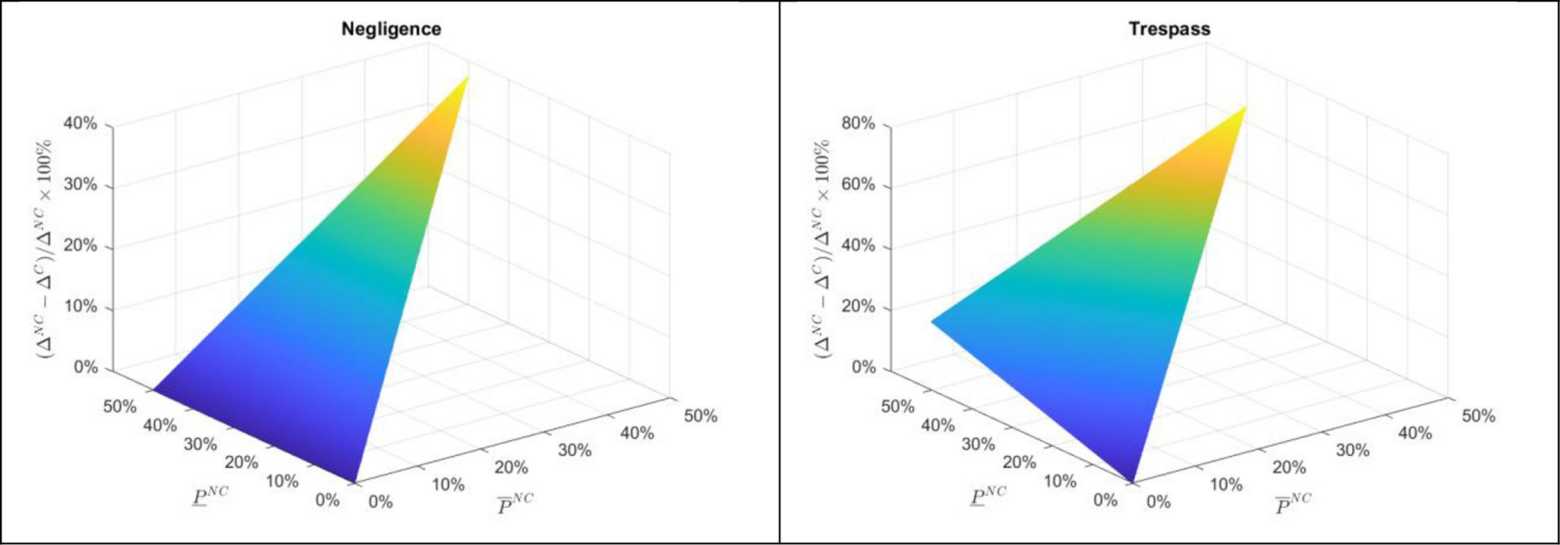
Example of the necessary percentage profit increase for applicators from non-compliance with pesticide application regulations under negligence and trespass.

Considering a range of probabilities of damages occurring under non-compliance (${\bar{P}}^{NC},{\underline{P}}^{NC}\in [0,0.5]$), Fig 4 presents the minimum percentage increase in profit, $\frac{\left(\Delta^{NC}-\Delta^{C}\right)}{\Delta^{NC}}*100\%$, required for an applicator to optimally choose non-compliance under negligence vs. trespass. As can be seen in both the negligence and trespass plots, the required increase in profit under non-compliance is increasing in the probability of high damages under non-compliance, ${\bar{P}}^{NC}$, as this increases the likelihood of a lawsuit being filed. However, as demonstrated in the theoretical section, the incentive to comply with labeling regulations under negligence is unaffected by the probability of low damages under non-compliance whereas under trespass the required profit threshold increases. Further, as also demonstrated in the theoretical section, for any given level of damage probabilities, negligence requires a lower reward to the applicator for non-compliance and, as a result, will result in less compliance.

**Fig 5:**
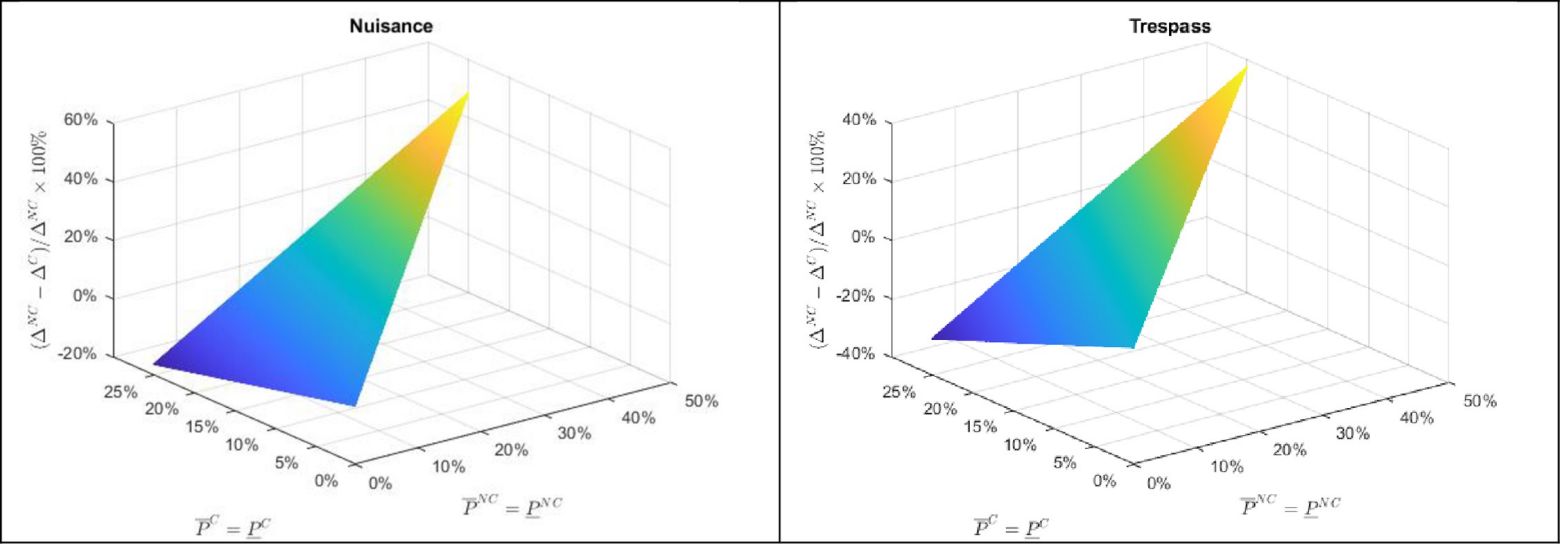
Example of the necessary percentage profit increase for applicators from non-compliance with pesticide application regulations under nuisance and trespass.

To further illustrate the difference in incentives for compliance under the alternative legal structures, consider a relaxation of the probability of damages under compliance being zero. For simplicity of illustration, suppose that the probability of low and high damages are equivalent for both the case of compliance (${\bar{P}}^{C}={\underline{P}}^{C}\in [0,0.10]$) and non-compliance (${\bar{P}}^{NC}={\underline{P}}^{NC}\in [0,0.5]$). As a neighboring farmer under negligence will only bring a lawsuit in the case of high damages by a non-compliant applicator, the incentive for an applicator to be non-compliant is unchanged from Fig 4 for a positive probability of damages by a compliant applicator. However, under trespass and nuisance, as shown in Fig 5, a non-zero probability of damages under compliance alters the incentive for an applicator to follow pesticide application regulations. As the probability of damages from pesticide drift under compliant application increases, the incentive for an applicator to comply with pesticide label prescriptions decreases. That is, since under nuisance and trespass an applicator may be held financially liable for damages even when following all proscribed application rules, if there is indeed a non-zero probability of damages under compliance, then this would erode some of the incentive for an applicator to be compliant. This, in particular, highlights the importance of confidence the EPA and pesticide manufactures to appropriately test and label products to offer confidence to growers that following labels and regulations will not result in pesticide drift and damages.

## 4. Discussion and conclusion

As state agencies and the EPA continue to manage the dicamba situation, losses from drift remain a heightened possibility for farmers across the country. The path for legal remedy to these losses can be confusing, if not impossible, for many farmers to navigate. If state governments seek to improve this system, they will realize that each tort presents different tradeoffs to groups within the farming industry. Depending on which tort is permitted for use as a cause of action in drift cases, the tradeoffs stand to improve certain groups and worsen others. These groups will be called the ‘winners’ and the ‘losers,’ respectively, under the specific tort model. Before a state can make a reasonable change to their jurisprudence system, it needs to have clear priorities on which groups should finish as winners as a result of the change. Therefore, this paper will conclude with the identification of the winners and losers under each tort model. Additionally, the most likely process with which the tort would be implemented as a viable cause of action will also be included in order to avoid potential complications related to policy changes.

### 4.1. Negligence

A negligence system is established when the neighboring farmer cannot use nuisance or trespass to recover drift losses. A negligence system can thus be reverse engineered simply by precluding the implementation of either a nuisance or trespass system. Specifically, the state must have a strong right-to-farm act that broadly limits nuisance actions against farms. The state court system must also reject the substantial damages requirement for trespass which would allow intangible invasions such as pesticide drift to qualify as an actionable trespass. If those two steps are satisfied, then a state has limited the pool of viable drift-related actions to only those that include non-compliant applicators.

Under a negligence system, farmers who use HT crop systems can easily be identified as winners. Farmers who use HT systems face the lowest probability of being sued for drift resulting from their herbicide applications. These farmers can only be sued if their applications are found to be non-compliant. Even then, non-compliant applicators under negligence face weakly superior outcomes than they do under trespass and nuisance. Furthermore, HT crop fields face a decreased likelihood of being damaged from drift due to their genetic immunity to at least one if not multiple herbicides (e.g., dicamba-tolerant crops are often also tolerant to glyphosate). The combination of a relatively low probability of being sued and a similarly low probability of suffering drift damage allows farmers of HT crops under a negligence system to have their highest expected profits out of all three models. The tradeoffs offered to HT applicators would cause an increase in the demand for HT crop systems in a negligence system. Therefore, the pesticide companies that produce HT crop systems are also identified as winners under negligence.

With respect to the losers in a negligence system, farmers who do not use the same pesticide as their neighbors face the most challenging path out of all three models to recovering drift losses. This aspect holds true for both farmers of annual, perennial, and Certified Organic crops because the surrounding applicators face the lowest probability of being sued under negligence. Making matters even worse for perennial and Certified Organic farmers, injunctions are not common under negligence systems, even with non-compliant applicators. Therefore, applicators have the lowest incentive to avoid volatile pesticide use. Without significant disincentives to volatile pesticides, an applicator could theoretically apply the same volatile pesticide over consecutive seasons and repeatedly wipe out parts of the neighboring farmer’s cropland. If these applications were compliant, the neighboring farmer would not even be able to recover their losses from the drift.

Interestingly, the second group of losers from the use of negligence is comprised of the EPA and state agencies. These regulatory agencies establish the requirements for a compliant application. Applicators obtain legal immunity from negligence lawsuits by performing compliant applications. This aspect of a negligence system emphasizes the need for regulators to make extremely accurate judgments in their rule-making on pesticide applications. Repeated updates to dicamba regulations over multiple seasons support the argument that adherence to these regulations is not sufficient to prevent the occurrence of pesticide drift [4]. There is also notable case law that gives additional examples of compliant applications that nonetheless resulted in drift damage on neighboring farms [8, 52].

Though *Bates v*. *Dow Agrisciences LLC* (2005) focused largely on state versus federal pesticide regulations, the Supreme Court emphasized the importance of tort litigation in pesticide regulation several times, noting that “tort suits can serve as a catalyst in this process [of improving pesticide labels]” [36 p451]. In *Bates*, the Supreme Court also cited *Ferebee v*. *Chevron Chemical Co*. (1984) which mentioned the possibility that tort litigation could bring the EPA’s attention to changes that need to be made to pesticide labels in light of new information [36, 62]. Without litigation that involves compliant applications, the EPA might not even learn about drift from such incidents thus further hindering regulators’ ability to write and potentially rewrite adequate regulations.

### 4.2. Trespass

Trespass can be incorporated as a civil remedy for drift losses through a precedential court ruling that recognizes pesticide drift as a legitimate cause of action under trespass theory [46]. Though courts in many states have thus far declined to set such a precedent, many of these courts have not explicitly ruled it out [63]. Oftentimes, these courts that initially rejected a trespass label on intangible invasions assert conditions under which said claims could succeed. Generally, the missing element from plaintiffs in those failed trespass cases is the demonstration of damages that occurred as a result of the intangible invasion. Though states vary on the specific legal category of damage that they desire to satisfy this requirement (e.g., substantial damages to *a res*, physical damage, etc.), crop losses from pesticide drift will generally meet any of these requirements if properly pleaded [7]. Therefore, for trespass to become a viable cause of action in drift suits, a state will need at least one farmer suffering losses in yield from drift to adequately plead his losses to a judge who is willing to allow a trespass case under the circumstances. This way, the state will set a new precedent that allows trespass to be used in cases of pesticide drift.

Perennial farmers and USDA Certified Organic farmers are identified as winners under trespass. These two groups of farmers face a distinct risk from pesticide drift in that they stand to lose both present and future crop yield from drift losses. As noted alongside the model for trespass, these two groups of farmers place the highest values on enjoining their neighbors from future pesticide applications. The trespass model is unique from nuisance and negligence in that the neighboring farmer can enjoin an applicator regardless of the applicator’s compliance. Given the increased risk they face drift along with the ease in which an injunction can be obtained, perennial farmers and USDA Certified Organic farmers are most satisfied under a trespass system.

Farmers of annual crops have a lower value for the injunction, given that their losses drift are usually only felt for one season. In cases of drift, annual crop farmers are mainly concerned with the recovery of their losses for that season, which is possible under trespass. Therefore, they are generally just as satisfied with trespass as they are with nuisance and are considered winners under both systems. Extreme cases of drift occurrences throughout multiple seasons may lead these farmers to place a higher value on the injunction though. Therefore, similar to the perennial and Certified Organic farmers, farmers of annual crops are also slightly more satisfied under a trespass system than a nuisance system. Still, it is worth noting that the gap between satisfaction levels with both tort systems is larger for the perennial and Certified Organic farmers.

Farmers that utilize HT crop systems can be viewed as losers under trespass. These farmers can broadly spray herbicides across their fields when their crops (and likely their neighbors’ crops) are growing in the field. While this is the main selling point of HT crop systems, the increased number of herbicide applications entails more opportunities for drift to occur. Due to the fact that HT crops cannot be sold with a price premium similar to that of their non-HT counterparts (e.g., Non-GMO, Certified Organic, etc.), farmers of HT crops maintain profitability through high yield rates that are made possible by additional herbicide applications. The increased number of opportunities for drift to occur along with their financial reliance on the additional applications mean that farmers of HT crops in a trespass system face both an increased likelihood of being sued and a more significant harm from the resulting injunction. The dangers posed by farming HT crops in a trespass system would also be felt by the companies who sell HT seeds and their corresponding herbicides, thus making them losers under trespass as well.

Until this point, it has likely become apparent that the winners under negligence were also losers under trespass and vice versa. When it comes to the discussion of how pesticide regulators fare under a trespass model, this trend ceases to continue. Though the EPA and state agencies will be listed as winners under nuisance, it is difficult to consider these regulatory agencies as winners under trespass because of the unpredictability of the language of the injunction. As noted in the first key assumption of the model, courts write injunctions at their own discretion and there is significant variation in how courts have historically written injunctions [30, 49]. The recent issues with dicamba provides a potential scenario in which judges in a trespass system enjoin numerous compliant farmers from the use of dicamba to protect neighboring farmers’ property rights. In such a scenario, the judges would be considering the scientific data that suggests dicamba can drift over a mile from the targeted crop [26]. While negligence poses issues for pesticide regulators because they might not become aware of unfiled drift cases, trespass poses issues for pesticide regulators because they might not get the opportunity to fix issues with the original label rules before enough applicators simply move to a new pesticide. Despite this weakness of trespass for the EPA and state agencies, the issues with dicamba drift did not result in any large abandonment of the use of the herbicide but instead changes in both federal and state dicamba regulations that may be considered responsible for subsequent decreases in drift [1]. Therefore, the EPA and state agencies are not labeled as winners or losers under the trespass model.

### 4.3. Nuisance

As stated in the legal discussion, pesticide drift is a textbook example of a nuisance [51]. But in most states, nuisance is broadly precluded as a remedy for pesticide drift due to right-to-farm acts [58]. Consequently, the means for allowing nuisance in drift cases entails amending right-to-farm acts. Specifically, states would need to amend their right-to-farm acts to include language such as that in Nebraska’s right-to-farm act which allows nuisance suits to continue when the offending farm would have been considered a nuisance when it commenced operations [53]. This option is fairly lenient to pesticide applicators as it requires the neighboring farmer to have been farming the land for at least as long as the applicator has been farming their land. Still, it does provide a pathway for nuisance to be used as a cause of action in cases of pesticide drift [52]. Given that most farmland has been under continuous agricultural operation for decades, this requirement would likely not pose an issue for most potential plaintiffs.

Should a state choose to promote the use of nuisance, two major winners can be identified. As noted in the previous subsection, farmers of annual crops are the first group of winners under nuisance. For this group, nuisance provides a desirable remedy as its frequent use of monetary damages means that they simply recover their financial losses (minus legal expenses, $\bar{D}-L$) from drift, regardless of the applicator’s compliance. In the event of applicator non-compliance, these farmers could also factor the value of an injunction in their decision of whether or not to file lawsuit. As mentioned in the previous subsection, farmers of annual crops likely place a low value on the injunction, meaning that nuisance and trespass offer essentially identical outcomes to this group. Still, injunctions are possible under nuisance if the applicator was non-compliant, which could provide some benefit to farmers of annual crops who for whatever reason place a higher value on the injunction.

Farmers of perennial crops (e.g., peach orchard, blueberry vineyard, etc.) are excluded from this first group because their losses from just one incident of drift will continue for seasons after the incident as new trees, vines, etc. take time to become as productive as the previous ones. While the possibility of an injunction against a non-compliant applicator is beneficial for these farmers, the alleged drift issues with compliant dicamba applications in 2017 and 2018 revealed the shortcomings of limiting injunctions to only non-compliant applications. The sheer magnitude of drift incidents in those years makes nuisance a risky system at best for perennial farmers given their high value of the injunction. For similar reasoning, USDA Certified Organic farmers cannot be accurately labeled as winners under nuisance either. Organic farmers must restart the three year-certification process if drift causes them to exceed the 5% threshold for synthetic pesticide residue [64]. During the certification process, these farmers are required to follow organic farming procedures but cannot market their products as organic [65]. The assumption that a court could grant the future value of these losses is uncertain, and the benefits of nuisance to both perennial farmers and Certified Organic farmers could be an appealing topic for future research [48].

The second group of winners under the nuisance model is comprised of the EPA and state agencies. Unlike negligence, nuisance produces similar outcomes for the neighboring farmer regardless of applicator compliance, with the exception being the injunction granted in situations of applicator noncompliance. The requirements for a compliant application are established by the EPA and state agencies. Under negligence, a potential disqualifier for the neighboring farmer to recover damages is whether or not the applicator obeyed the necessary regulations. This aspect of a negligence system emphasizes the need for regulators to make extremely accurate judgments in their work. Repeated updates to dicamba regulations over multiple seasons supports the argument that adherence to these regulations is not sufficient to prevent drift occurrence [4]. The implementation of a nuisance system could potentially reduce the neighboring farmers’ scrutiny on regulators, as they would have the ability to recover drift-related losses regardless of their neighbor’s adherence to (often imperfect) regulations. Unlike trespass, the nuisance model does not incorporate injunctions against compliant applicators, which could similarly reduce the applicators’ scrutiny of the regulatory bodies since they cannot be enjoined for following the necessary regulations.

After discussing the winners in a nuisance system, it should be unsurprising that the losers in a nuisance system are farmers of HT crops and consequently the companies that produce these seeds and their corresponding herbicides. These farmers are considered losers under nuisance for much of the same reasoning that earned them loser status under trespass, given their reliance on applications combined with the fact that applicators in both models earned relatively low profits and were sued more often than under negligence. Due to the lack of an injunction under the compliance setting of nuisance, farmers of HT crops are weakly superior under nuisance than under trespass. Still, the financial disincentive from the payment of monetary damages could induce changes in pesticide applications that somewhat resemble changes mandated by an injunction.
